# Thoughts on the Nature and Treatment of Several Severe Diseases of the Human Body

**Published:** 1847-07

**Authors:** 


### Art. IX.

*Thoughts on the Nature and Treatment of several Severe Diseases of the Human Body.* By E. J. Seymour, m.d., f.r.s., &c. In two Volumes. Vol. I.—*London,* 1847. 8vo, pp. 260.

Incredible as it may appear to those who shall attempt to follow us in the same path, it is, nevertheless, true, that we have gone over the volume before us three times—have literally read it thrice from beginning to end: such are the painful but imperative duties sometimes imposed on the conscientious reviewer! We had several reasons for the performance of this feat, the glory of which, we venture to believe, will for ever remain exclusively our own. In the first place, we were desirous of knowing thoroughly the "thoughts" of a man who had been long recognised as one of the busy and fashionable physicians of London, and who, over and over again, boasts, in the volume before us, of his great opportunities and exertions past and present. "These 'thoughts' (he tells us) have been put on paper under unceasing professional occupation" (Preface); "after more than a quarter of a century in the daily exercise of my profession" (p. 39); "in St. George's Hospital, during eighteen years' most assiduous duty" (p. 26); "a most diligent observer of post-mortem examinations, and with great opportunities for more than a quarter of a century" (p. 66). In the second place, all the diseases treated of being of daily occurrence, and consequently of great importance, we were determined that our readers should not be deprived of any information that might be in any degree useful in practice, through any disagreeable influence created in our mind by the form and mode in which it was conveyed. In the third place, we will candidly admit that we took up Dr. Seymour's volume with a slight prepossession against the author, originating in the popular reputation attaching to his name, of his being the only practical physician in Europe, under sixty years of age, who derided auscultation and set the stethoscope at nought. Having ourselves, in common with every other physician of Dr. Seymour's standing but Dr. Seymour himself, had the unquestioned and unquestionable physical truths of auscultation established as part and parcel of our mental furniture for more than twenty years, feeling it just as easy to doubt of the existence of the organs with which the phenomena of auscultation are associated as of the existence of these phenomena, or of the positive diagnostical significance of many of them; knowing, moreover, that it is impossible to do justice to one's profession, to one's self, or to the victims of many pectoral diseases, without the knowledge of these truths, and that they were, furthermore, of easy acquisition; we found it hard to conceive how any man of sound mind, of an average standard of intellect, and with even a moderate amount of moral endowment, could think as Dr. Seymour thought, and act as Dr. Seymour acted; and, consequently, we felt some difficulty in believing that Dr. Seymour could produce a medical book of any sort worthy the notice of his brethren. But this being merely *à priori* reasoning which, though generally just, might be exceptionally false, we determined that any prejudice thence arising should not be permitted to warp the judgment. A fourth reason for bestowing unusual attention on Dr. Seymour's book, originated in the expectation of finding a source of interest growing out of the very peculiarity just named as a defect. Looking to the mental qualities requisite to raise and keep a man "in that bad eminence" which Dr. Seymour had attained—an eminence which certainly entitles him to be reckoned the boldest physician of his time—we thought we would be sure of meeting in his practice with boldness at least, if not originality, and might thus find metal more attractive than meets us in pages which never think of catching a grace beyond the reach of ordinary art. Lastly, we found many passages of the book so hard to understand, from the singular style in which the greater part is written, that we were compelled to go over it more than once for the mere purpose of coming at the author's meaning.

We regret to state that the result of all this extraordinary labour and attention is far from satisfactory; inasmuch as we ourselves have obtained nothing which we regard as adequate to repay us personally for our trouble, and only a scanty gleaning of very ordinary materials which we can communicate to our younger readers as worthy of their notice. Indeed, the perusal of this book has been to us, in many ways, a very painful task : among other consequences, it has given a heavy blow and great discouragement to more than one of certain cheering professional glorifications in which our contented and sanguine temper has often led us to indulge. For example—it has been a favorite theme with us, in talking over the subject of professional prospects with our younger brethren, to maintain the proposition that superior talent, and learning, and professional knowledge, *ceteris paribus*, are sure to lead to superior success—we mean worldly success—in practice; and *vice versâ;* and in proof of this, we have been accustomed to point to the character and actual position of all, or almost all our physicians and surgeons most estimated and best remunerated by the public. "By their fruits (we have said) ye shall know them ;" and referring to the works that have done most honour to British medical science in our day, we have had to name as their authors the very men who stand highest in the world's esteem. It might be invidious to give names in illustration; but we believe it will be found that taking the twenty or thirty physicians and surgeons who have the largest practices in London, they are really, with two or three exceptions, the very men whom Science would place where they now stand. At any rate, we should find that the most eminent of them who have given books to the world, have shown themselves in them to be well entitled to take their place in the front rank of educated and learned men. To refer to a few of the works of our senior physicians only that we have had occasion within these few years to notice in this Journal,—where shall we find in our medical literature writings more correct and scholarlike than those of Holland, Watson, Latham, Clark, Ferguson, Bright, Prout, Paris, &c. &c.?

The volume on our table, we lament to say, is of a very different stamp. It is one which, to speak mildly, it is neither creditable to a physician to have written, nor creditable to medical literature in the nineteenth century to have given forth. It is not enough to say that the book is written in a style that is careless, slovenly, and rude, often obscure, sometimes unintelligible; a considerable portion of it seems written in utter disregard of the ordinary collocation and relation of words and phrases, and not a little of it is actually ungrammatical and un-English. It would be waste of time to adduce instances of these defects on purpose; we doubt not most of them will be exemplified in a greater or less degree in the extracts we shall have occasion to make with other views; and we venture to say that no reader can turn over three consecutive pages of the volume without admitting that our statement is not beyond the truth. Nor are the demerits of the production confined to the style merely. A considerable portion of it is really a surprising mass of confusion and bungle, without order, arrangement, or consequence; facts and opinions, observations and conjectures, the author's and other people's notions, the past and present, the right, the wrong, truisms and transcendentalisms, being all jumbled together in most admired disorder. To analyse such a work, so as to exhibit anything like a regular epitome or even outline of its contents, will be admitted to be no easy task, and is one we should never have thought of undertaking, but from the position and standing of the author. Nor, indeed, shall we even now attempt to give a regular analysis of the book, but shall content ourselves principally with noticing, as we go over the pages, what strikes us as calculated to convey information of more or less importance to our readers, whether that information be of a positive or negative, of a commendatory or a monitory stamp.

It would, indeed, be most strange—more strange even than such a wonder as this book is—if two hundred and sixty pages could be written by "a physician and formerly a teacher," after the experience of "more than a quarter of a century," without containing something not generally known, that might be of value to the younger practitioner. But even here, we must content ourselves with very moderate doings, and must be excused for so contenting ourselves; the unlucky schoolboy who, after losing himself and frightening himself in the thicket or the quagmire, has just got into fair daylight and beaten tracks, must not be expected to have a rich stock of eggs or nuts to show when he gets home.

I. The first of the "Severe Diseases" respecting which Dr. Seymour gives us his "thoughts" in this his first volume are, DISEASES OF THE STOMACH. These he proposes to treat under the head of their more prominent symptoms, as pain, vomiting, &c.; and he gives his reasons for so doing, with his wonted lucidity and elegance, in the following sentences which constitute the opening paragraph of his book:

"At first I wished to have made a distinction between disorder and disease of the stomach; but one of these appears to me to lead, when neglected, so much into the other, that I shall speak of all under one head. There are also two modes of describing the different diseases: one is pathologically, which leads to obscurity, some pedantry, and much difficulty in the adaptation of remedies; and as my only object is practical advantage, I prefer the second, which begins with what is obvious, and proceeds with what is difficult and obscure to the observation in general. I proceed, then, to speak of by far the most obvious and distressing symptom in these troublesome disorders, though often not present in the most serious ones—viz. pain in the stomach." (pp. 1-2.)

The whole essay, which extends to seventy pages, is just such a performance as this introduction prefigures. It contains nothing that is new, and represents the old things in forms that are much less attractive and intelligible than we have been accustomed to meet with them in scores of preceding treatises on this interminable theme. Dr. Pemberton's slim volume, published some thirty years since, will convey much more information to the junior practitioner than all Dr. Seymour's whirligig lucubrations. The commonest remedies, rhubarb, magnesia, soda, bismuth, hydrocyanic acid, &c., prescribed in the commonest combinations, yet paraded in all the display of *Recipes,* are the staple of this part of the book.

The only thing unusual in the treatment of *Cardialgia* which we meet with is the large doses of opium recommended in certain cases. Dr. Seymour gives a grain three times a day, and mentions a case (p. 9) where this was continued for four weeks. No doubt this is true as regards this individual case, but we have no hesitation in saying that such medication as this will generally be found not merely ineffective, but most injurious. Opium has always been a favorite remedy with empirical and heroic practitioners, and we shall afterwards see that it is especially patronised by Dr. Seymour.

The milder cases of *Pyrosis* are said to be easily cured as follows:

"Gr. v of Pulv. Kino Comp. given three times in the day, and the bowels kept open by enemata or aloetic purgatives, speedily cures the disease, especially if combined with rhubarb, &c. &c. and a diet principally consisting of poultry, butcher's meat (once in the day), eggs, potted meat, curried food, and weak brandy and water as a beverage." (p. 10.)

Does the author give this diet as in any way peculiar?—We are glad to see, that in such cases he discountenances the use of "calomel and purgatives," informing us that, when so treated, they "became greatly protracted as to time and severity" (p. 10),—protracted as to severity! Under the date of 1843-4, he gives a case which is a very instructive one indeed. It was a case of stomach disorder in a lady, marked by vomiting, &c. "I repeatedly *asked her* if, by putting her hand on the pit of the stomach, she could discover any hardness or lump. The answer was: Certainly not." Some nine months after this she consulted Dr. Seymour again, with aggravation of the old symptoms, and with the addition of "an extreme sense of debility." To the same *queries* as to the state of the epigastrium, she replied as before:

"The patient became worse. About three weeks after this meeting, she had a fainting fit, and when summoned I found her in bed. This enabled me to examine the abdomen, and greatly was I shocked to find, not one tumour, but a number of tumours, varying from the size of a pigeon's egg to that of a hen's egg, occupying the whole epigastrium and the left hypochondrium—fungoid disease! I went home, resolving nothing should ever deter me from examining the stomach, especially before giving an opinion, in future." (p. 12.)

The patient died of course. Now we cannot help expressing some surprise that Dr. Seymour should have waited till the year 1844 to learn the necessity of examining the abdomen in all such doubtful cases, or that he should not have here been "enabled" to do so, until he accidentally found his patient in bed. This is surely not the kind of lessons he taught his pupils when "formerly a teacher."

The following elegant paragraph introduces the subject of gall-stone; and we may say that it completes it also, as we have scarcely anything else on the subject, except references to two cases which seem to be introduced—the one to show that the author was "Physician to the Asylum for the Recovery of Health, in the year 1827," and the other to record the unique fact of an old lady who to relieve pain "applied bottles heated to such a degree as to *burn the muscles* of the part." (p. 16.) Burn the muscles! Why, the bottle must have been not only red hot but solid or filled with solid matter, to retain heat sufficiently long to effect this.

"The next most obvious and most frequent case is where the pain in the stomach or at the scrobiculus cordis is indicative of gall-stone; either the passage of a gall-stone alone along the hepatic duct, the cystic duct, or the ductus communis; or even where there are a very large number of these concretions in the gall-bladder, so that, during the operation of digestion, any change made in its situation produces tension of the gall-bladder itself. The pain, in either of these cases, is similar. There is most violent pain at the scrobiculus cordis, shooting through to the back; pain as of grinding the part is felt. The pain is so intense that it appears absolutely impossible that it should long be borne; nevertheless, the pulse is never quick, and often preternaturally slow, as 50 or 60 in a minute, rarely above the latter. Sometimes this intense pain is accompanied by vomiting, but not always. However severe, it ordinarily disappears suddenly, as if by magic, and the patient, allowing for languor, is nearly as well as usual.

"When the calculus has entered the duct, whether it gradually proceeds forward or falls back, the pain is succeeded by a greater or less degree of jaundice, which immediately marks the disease; then, of course, the excretions are pale, often as white as chalk, while the urine is of the colour of porter; and this state continues until the concretion escapes into the bowel. Still there may be obstruction from a gall-stone, and subsequent intense pain at the pit of the stomach, tingling or itching of the skin, vomiting, while the urine is of the natural colour, and the fæces with the usual proportion of bile. In this case the calculus is in the cystic duct." (pp. 14-5.)

In the next fifteen pages we have an account of chronic ulcer of the stomach, perforation of this organ, and hæmatemesis, all mixed and intermixed in so strange a manner that it becomes almost impossible to find out to what particular disease individual observations or individual remedies apply. Here, also, the author ventures to leave the only path he should attempt to tread, and gives us some general statistics respecting the degree of prevalence of perforation of the stomach in the two sexes. We are disposed to agree with him, however, in believing that "there is no preponderating difference in either sex as to predisposition to this terrible disease." (p. 30.)

In the middle of this discussion the author, *more suo*, breaks out into quite a different theme: here it is a discourse on "diet in general;" and though he says he can "scarcely help incurring blame" for his opinions, we must say that they appear to us the least blameable part of his book. With the common-sense views of the following extracts, we entirely agree, and have the more pleasure in transcribing them because they are really good English—and do not, we believe, contain more than a single grammatical error.

"Other persons insist, when living in London, and never quitting their arduous duties, that they are free from all impeachment of irregularity, provided, as they say, they only eat meat, beef, mutton, and poultry; never sweets, entremets, jelly, cream, and the like; never eat pastry, not even apple-pie, and live in a delightful dream of civic anchoritism. These persons take no exercise, sit up late, often in heated rooms; their hearty meal of meat overcharges their blood (happy had it been divided with a little fruit jelly, or baked fruit, or even the anathematised apple-pie); they get heated, feverish, pains in the joints, pains in the back, and at length gout,—wondering where it can come from, as they never indulge!

"In speaking of health, the great advantage is to be sought from regular exercise, and in London life, if possible, on horseback; and great moderation. But if general rules be difficult, what shall we say to partial ones—to the unhappy hypochondriac, who sees poison in all that is wholesome, and has some book on diet to look at *à la dérobée*, to sustain him in his imaginary distresses? Is it a cause or an effect, that persons dreadfully sensitive about what is wholesome are always weak and sickly?

"If we watch, as I have watched persons on this subject, where is the agreement? or, what is more, from what is our experience to be derived? Some stomachs cannot bear nor digest animal food, as beef and mutton. To these, dishes cooked in the French fashion, where food is much subdivided, can be taken without pain, and with comfort. To some, fish, even those which are thought to be most injurious, give no pain; salmon, eels, mackerel, are all eaten without trouble, where mutton chops and beef-steaks give pain. In another case the first-named aliments are productive of sickness, pain, bad taste in the mouth, and all the annoyance of what is called bilious derangement ........

"How, then, are we to judge? I have been much struck by the facility, in illness, and in delicate constitutions, with which food much desired by the stomach is digested. Should not what I may call the instinct of this viscus be a little more consulted? and if there be a strong feeling or desire on this point, may it not be advantageously gratified in defiance of arbitrary rules? Every day, in extensive practice, we may be answered in the affirmative!

"Unhappily writers on this subject recommend from their own feelings or prejudices. A healthy hearty man recommends what he finds keeps himself in excellent health: while one with a weaker stomach cannot help conveying his ideas on the subject, which are very different. A man accustomed to the fare of a hot country like India, cannot manage the diet of a cold country, where great and active exercise is taken. The endeavour to bring an Indian to the wholesome food of an English farmer, would be to enact the old story of the man who brought his horse to live on a straw, and—he died.

"The whole question resolves itself, except in actual disease (fever, bowel complaints, violent cough, expectoration of blood, consumption, and diabetes, &c. &c.), into one small word,—moderation. To man in health, and in many diseases, all is comprised in that one word, moderation. Take what your inclination shows you to be desirable when in health, only avoiding what is manifestly injurious from your own experience. In illness nearly a similar rule may apply. A man in a fever could not be made to eat beef; and it is only in convalescence, or recovery from recent illness, that a man must be obliged to listen to lessons of absolute self-negation!" (pp. 32-4.)

Although our author tells us that, in this part of his work, he has "followed the natural course as far as the mind can be carried from one immediate subject to another," (p. 34,) and although we think we understand what he means when he says so, we must confess that we find it as hard work to keep up with him as if his "course" were the most unnatural in the world, and his subjects were anything but "immediate." We must, therefore, be content to pick our way the best we can, and hop, skip, and jump with him backwards and forwards as he pleases. After his digression on diet, he jumps back to *vomiting;* of which he describes numerous varieties. This is one:

"Vomiting is also a prominent symptom in some cases of phthisis pulmonalis, —nay, in some rare instances, I have seen it so severe, as to draw the patient's attention and that of his friends entirely away from the real disease. Vomiting in phthisis pulmonalis, occurring almost always after coughing, if it arise early in the disease, is the proof of a severe and rapid form of it; if late, *it betokens that large collections of matter are locked up in the lungs*, that is, have not yet found an outlet through the larger branches of the bronchi. I have had occasion to point out this symptom during life, and to illustrate its cause after death, to the pupils of the hospital." (p. 41.)

In illustration of this statement a case is given, of what Dr. S. calls "phthisis pulmonalis of the languid kind," and which exhibits just such blundering as must often, of necessity, follow the rejection of auscultation in practice.

"Sarsaparilla, followed by gentle tonics, and afterwards quina, with a mild and nutritious diet, and medicine to allay the cough, *appeared* at the end of two months *to have effected a very material change.* The sweats had disappeared, there was increased strength, and *it was proposed to the patient's anxious friends that, to avoid the March winds, he should be removed to Hastings.* The last consultation, before this plan was to be carried into effect, took place between the late Dr. Nevinson and myself. I remarked—'Well, he is wonderfully improved to all appearance, but I never can think this change otherwise than delusive so long as he continues to vomit at the conclusion of a fit of coughing.' 'It is a very disagreeable symptom,' said Dr. N., 'but it is the only one?' About eight and forty hours after this conversation a vomica gave way in a fit of coughing, and more than a pint of purulent matter was brought up. I say a pint, for it nearly filled one of the large finger-glasses used at table. The patient died four days after this event, the more violent symptoms of hectic immediately following the discharge." (pp. 42-3.)

How different, in all probability, would have been the prognosis here, and also the advice, had the physicians only condescended to percuss the patient's chest or apply the stethoscope but for a few minutes! And we could adduce several other illustrations of the same kind from this volume, all showing the miserable shifts to which the author has reduced himself by discarding auscultation from his practice. Without stopping to inquire what Dr. Seymour means by a "vomica," and thinking it very probable that the pathological state in the case in question was quite different from what he supposed it to be, is it not melancholy to see a physician in these times, looking to a mere sympathetic symptom like vomiting, for evidence (conjectural and circumstantial at best) of a great organic change in a vital organ, when positive demonstration of its existence and amount could be at once obtained? Dr. Seymour, like the late Sir Matthew Tierney, a name of authority in the fashionable world in the days "when George the Fourth was King," though now forgotten, no doubt considers the practice of auscultation as unscientific and undignified, fit only for "low mechanicals," and debasing the noble profession of physic. To simple and humble-minded men like ourselves, however, this flying from what is easy, clear, and certain, to what is difficult, obscure, and problematical, seems egregious trifling: and Dr. Seymour's diagnosis of what he calls Vomica, by means of the symptom Vomiting, reminds us of the super-scientific tailors of Laputa, who, disdaining the vulgar modes of fashioning garments by direct *mensuration* and *palpation*, took measure of their patients by the quadrant and astrolabe—a highly ingenious proceeding, doubtless, but resulting, as the veracious historian tells us, in marvellously ill-fitting clothes.

Dr. Seymour says that there is a peculiar kind of vomiting, which is pathognomonic of the disease which has been called by Dr. Baron, "tuberculated accretion of serous membranes," or chronic tubercular peritonitis. This vomiting is of "a green so dark that it is only to be compared with that which the sea acquires at great depths ; or blue, as intense nearly as that of indigo ; deep green when regarded in one way, blue in the other." (p. 47.) He does not say that it is always present, but only "when it does occur, it is diagnostic of disease." This affection, he truly tells us, in his own odd way, is "by no means uncommon in young people, especially females, and not very uncommon in youthful adults" (p. 46) ; and we agree with him as to the mistake so commonly made of confounding it with disease of the mesenteric glands. We fear, however, that further experience will yet teach Dr. Seymour that his "peculiar vomiting" is not pathognomonic of this disease, but that it attends more than one other affection of the abdomen.

After what they have already seen, our readers will not be surprised to learn that the author brings into his book, under the head of diseases characterised by vomiting, *Asiatic cholera.* We think, however, they will hardly expect to read such platitudes as Dr. Seymour gives us on the treatment of this disease (and we extract *all* he says of Asiatic cholera) ; any more than he could expect such a specimen of the Mrs.-Gamp-school of pathology as the next extract presents ; all, too, written in the year of grace, 1847, and by a learned Fellow of the College of Physicians, and in what he terms "a paper purely practical." We need hardly call attention to the accuracy, elegance, and precision of style in which the information is conveyed : he that runs may read.

"I have spoken of a peculiar coloured vomiting, which, when it is present, distinguishes the disease known by the name of 'tuberculated accretion of the peritoneum ;' but in the most frightful disease with which modern physicians have become acquainted, Asiatic cholera, the vomiting which characterises it is entirely devoid of colour, like thin gruel, or water in which rice has been boiled ; and where it is tinged with bile it is considered to be a favorable symptom. Various modes, in the hands of different practitioners, have been used to stop this discharge or correct its quality. In India, it would appear that calomel and opium most frequently succeeded. (See a paper in the Medical and Chirurgical Transactions, communicated by the late Sir Gilbert Blane.) In the disease which visited Europe in the year 1830, and England in 1831, *great good was expected* from evacuating the stomach early by a mustard emetic ; others found more advantage from very small doses of Epsom salts, ℈ss. and magnesia, gr. v, with two or three minims of laudanum in a table-spoonful of water, every three or four hours, or oftener,—a mixture which has been found very useful in inordinate vomiting in the diseases already mentioned, and also in the dysentery and diarrhœa of India. It is very remarkable, that the very remedies which, on the first onset of the disease, were utterly useless, were found very beneficial *as the time became longer* and the disease progressed. In all the northern countries, and in our own, the same proportion of deaths occurred during the first month of the invasion of the disease. This diminished greatly as time advanced, and towards its disappearance the proportion of those who recovered, to those attacked, bore a very large ratio to the mortality which happened in the first month." (pp. 56-7.)

"The pain at the pit of the stomach [in chronic gastritis] is equally a symptom of distended liver ; and it is not easy to understand how a viscus supplied by the same vessels as those which supply the liver and spleen can be inflamed even for a short time without these viscera suffering. The stomach may, indeed, suffer from alterations in its peculiar secretions, especially in their chemical composition; but, that inflammation should occur in a chronic form, without affecting the neighbouring viscera, fed and nourished from the same trunk, seems difficult to believe." (p. 63.)

II. The section on Gout occupies nearly eighty pages, all of which we have gone over and over again, without being able to find any one new truth in pathology or practice, or an observation of any kind that could either justify or account for its publication. The "thoughts" are here less disturbed in their "natural course" than they were when the author was dealing with the multiform affections of the stomach; still they are very flighty and vagrant, and what is meant for ratiocination is not a whit more consequent or logical. The language seems not at all improved as the work advances, as our extracts will probably show. It is in the following strain that Dr. Seymour introduces his subject:

"Experience in my profession for more than twenty years has shown me many cases of gout in the classes of society where it is the most frequent in its various forms, whether of positive disease or constitutional disposition. The following remarks contain my observations as a physician, and formerly a teacher, on this frequent and most painful disease: still, to write observations on this subject appears to be the commencement of an undertaking more than ordinarily imprudent. The disease was well known to the ancient physicians, as I shall often have occasion to mention. In modern times, and in England alone, when I say there are upwards of 120 treatises on this disease (I speak very much below the real number), does it not appear presumptuous to add to this number, in the hope of saying anything new? I do not know that I can say anything new, but the arrangement of our present knowledge on the subject, our treatment and the reasons for that treatment, are not satisfactorily stated, in my own view of the case, concisely and for practical use. It is to supply this (temporary if it be) want, that having experience on this subject, I undertake the task of making the following observations. This disease was first known to dissolute society, where good living and indolence were mingled together, and it descended to the offspring of those who indulged; and as indulgence increased with luxury, so the disposition to transmit acquired disease was established. In fact, this disease was principally restricted to the rich and powerful of every age, and hence became the object of interest to physicians, and attracted their attention.........

"If, in the course of two thousand years, remedies, especially from the vegetable kingdom (which in such a space of time has undergone no alteration), have been recommended by the wisest of their day, have fallen into disrepute, buried or forgotten, have again been revived and used with great advantage, and perhaps this has happened a third time,—such a confirmation of their utility in different ages, under different theories, and in the habits of different nations, ought to make them acquire a greater stamp of value, rather than the contempt attributed to implements invented in an early and a savage state.........

"What is gout? More than two centuries have elapsed, during which men, versed in various kinds of learning, and while new discoveries were pouring in, afforded a satisfactory reply for the moment to this question without attempting to give a full answer." (pp. 71-5.)

"Gout is not *the result* of a natural state of things; nor do I believe it ever exists in a state of nature. It is the scourge of civilized life; and hence is mixed with the pleasures and enjoyments of the table, and the cares and exertions of ambition." (p. 142.)

We entirely agree with the author in thinking that for him, to "write observations on this subject is an undertaking more than ordinarily imprudent;" but we hope he really does not mean that this is only "the commencement" of the undertaking! We would also fain hope that however

" dissolute" our good society may be, we may continue to get on without any more of the "thoughts" or "observations" here "commenced," more especially as gout will always be an "object of interest to physicians *and* attract attention;" and we humbly trust—whatever Dr. Seymour's fears may be—that "the vegetable kingdom" which supplies us with our remedies, will "undergo no alteration" even "in the course of two thousand years," but, by continuing to yield colchicum, burdock, &c., may enable us to give "a satisfactory reply, though not a full answer" to all who shall seek reasons for the faith that is in us.

After a miserably meager, and very incorrect view of the established opinions respecting the pathology of gout, the author comes down to his own proper sphere—that of telling us what his own eyes have seen in ordinary life and common practice,—and gives us the following very good account of a first attack of gout. *O si sic omnia !*

"At a period generally between thirty and forty years of age, earlier if hereditary, later if arising for the first time, the patient, usually a good liver, of sedentary occupations, and who has suffered most frequently what is commonly called 'bilious complaints,'—that is, he has suffered much from dining out frequently, and eating and drinking largely,—has acid in the stomach, flatulence, and a fear, not of the quantity he eats, but of the quality : saying, that he is a most careful man, always eats plain things, mutton or beef; never tastes sweet things, never eats butter, and considers himself a paragon of care : but he does not say that he works all day and part of the night—perhaps in the House of Commons—eats late of fish and soup, and then heartily of *beef* and *mutton*, and potatoes, and porter, and being worn out, takes some wine afterwards. Well, all this is very well, if he rode six hours a day, or followed the hounds, or walked many hours. But the nutrition is constantly introduced, while the exhalation from the skin produced by exercise is wanting; the regularity of the alvine and renal excretions is all in abeyance. Such a man, between forty and fifty, having been low-spirited, flatulent, and unable to eat, with heartburn, is perhaps, after exposure to cold, perhaps after a late party, perhaps after sitting up late writing, much against his wishes, attacked about two o'clock in the morning with acute pain, acute enough to make him cry out, in one foot." (pp. 80-1.)

We cannot find a single novelty in the account Dr. Seymour gives of the treatment of gout, which he truly and quaintly tells us "has varied little since the earlier knowledge of the disease, *except from occasional violent practices*." It "consists in keeping the bowels open with warm and aromatic purgatives, in regulating the diet, and, during the fit, employing opiates to relieve pain, and colchicum to diminish inflammation." (p. 87.) He is a great advocate for colchicum, and combats the opinion of its being injurious to the constitution when used in moderation. "In *my opinion*, (he says) the wine of the root and the acetous extract, are the most frequently and effectually employed." p. 96. Dr. Seymour draws a just distinction between acquired and hereditary gout in regard to practice. The following extract may be useful to the young practitioner, as giving in a few words all that is essential in the treatment of a paroxysm—though, indeed, all his teachers must have told him the same thing long ago :

"Speaking, then, first of the gout which is acquired, or the case in which gout in the family is a very remote circumstance, the following is ordinarily the practice :—The bowels are to be freely opened with calomel, colocynth, and scammony (℞ Hydrarg. Chlorid. ; Pulv. Gummi Scamm. aa. gr. ij ; Extr. Colocynth. Comp. gr. iv. M. Ft. pilulæ, ij h. s. s.), and then the colchicum is to be given, about twenty drops of the wine twice daily, and at night three grains of the acetous extract, with five grains or more of Dover's powder, according to the *nightly pain:* this must be soothed at all events. This course may be continued. The purgative should be given every second or third night, and the patient kept on low diet; and in a first attack from six to ten days is the usual duration of the paroxysm. But where the attack (without marked hereditary taint) has often been repeated, it will be better to give a dose of colchicum only at bedtime, combined with an opiate; taking every alternate morning a dose of Cheltenham salts in *warm* water to obviate constipation. ℞ Vini Rad. Colchici, ʒss.; Solut. Acetatis Morphiæ, ♏xx (gr. ½); Aquæ Flor. Aurantii, ʒj; Aquæ Fontanæ, ʒx; Syrupi Aurantii, ʒj. M. Ft. haust. h. s. s. o. n. If, indeed, fever runs very high, with hot skin, pulse very quick, and great febrile distress, to the night draught may be added from twenty to thirty drops of the liquor antimonii tartarizat. with good effect. Variations of this treatment will generally meet all the difficulties of regular painful gout in the paroxysm; still, after several attacks, more must be thought of." (pp. 99-101.)

Dr. Seymour dwells much too little on the *preventive treatment* of gout, or the treatment in the intervals; although this is infinitely more important than that of the paroxysm. His practice is the common every-day routine, which is good enough as far as it goes, but very bad when it is considered as, in any degree, a substitute for that non-medicinal treatment which can alone lead to results of any substantial and permanent value. Here is the medicinal treatment; and it is really curious that the author could not tell us so simple a thing as this without puzzling us as to his meaning and his grammar, twice at least in his two sentences.

"The usual practice, and a most effectual one, at present, in the intervals of gout, is to endeavour so to act upon the stomach as to prevent the formation of acid, which first arising in the stomach, seems to point out the morbid condition of the secretions of the bowels, kidneys, and skin. For this purpose, various combinations of a fixed alkali with a vegetable bitter, some warm stomachic made gently purgative, seems to fulfil the intention of the ancient physicians with the alterations shown by modern chemistry. Thus, rhubarb in all its forms has been joined with soda or potash, and with cinnamon or ginger." (p. 130.)

To be sure, we have also a brief account of the effects of warm and a much longer one of the effects of cold bathing, (including the new system of Hydropathy) both of which are commended; but we desiderate that complete and combined system of action in regard to regimen and general hygiene, which can alone justify the confidence of physiological physicians. When treating of hydropathy, (in which Dr. Seymour seems to us to possess a reasonable, qualified confidence,) he makes the following sensible observations, which will remind some of our readers of the article in our 44th Number.

"Now let us look at the question as it applies in general, and as regards that class of society who are affected with gout. A man worn out with fatigue, and living in this close town,—perhaps an artist, with warm and vivid feeling, toiling all day, and finishing the evening with supper and spirits and water, and perhaps cigars,—becomes hypochondriacal, loses appetite, and loses spirits. He is recommended to a hydropathic institution in the country. He *must* remain there a fortnight or three weeks. He is at leisure; he uses the alternations of heat and cold; the skin acts plentifully; he has good wholesome diet, not in excess, meat and vegetables, in a beautiful country, with clear, pure air, and early rising, and cheerful society, &c., drinks water in considerable quantities, and no wine. Can anything be imagined more likely to restore his spirits, tone down his irritability, release him from anxious thoughts, at the very time that the novelty of the treatment makes him consider himself to be in some degree an object of observation? Can any one doubt that this must, in many cases, be useful?" (p. 141.)

We think not. But it is but justice to Dr. Seymour, as well as to ourselves, to add, that the cases must be carefully selected for this practice, and also, that the practice must be applied and modified according to the exigencies of individual cases, and the laws of physiology and pathology. Therefore our author justly adds to the above extract:

"But, had the seeker after health a diseased heart or diseased lungs without his knowledge, or repressed gout, diabetes, or diseased kidneys, or dropsy from diseased liver, would these effects have been produced? Certainly not. It will restore impaired and faded strength, and with effect exactly proportionate to the mental wear and tear of the sufferer, but nothing more; nay, it will do harm if urged beyond the circle of functional disorders." (pp. 141-2.)

III. The third section of Dr. Seymour's work, which occupies about eighty pages, has this lengthy, clumsy, and not very intelligible title prefixed to it, in capitals:

"On mental derangement, especially that form termed melancholia, or the alternation of melancholia with violence, termed lypomania, or suicidal madness, especially in reference to the treatment of this form of disease, by the employment of opiates, or of that class of medicines called sedatives."

Without stopping to inquire on what authority Dr. Seymour makes lypomania and suicidal mania one and the same disease—for we presume this is the meaning of his words,—or to criticise his subsequent observations on the nature of mania in general, we proceed at once to notice the principal feature of the essay, the use of opiates in insanity. We entirely agree with the author in thinking that where there exists no manifest organic affection of the brain, in "this the most afflicting disease in human nature," "we are bound to apply the resources of our art to its cure" (p. 153); that is, to try the effect of medicinal as well as moral means in its treatment. The great medicament in the author's opinion is opium. We ourselves believe also that it is a very valuable remedy in certain cases; but the difficulty is, to recognise the fit cases in practice. Had we found that Dr. Seymour had here done what he professes to do, and probably thinks he has done, viz. "to point out the cases in which such treatment is or is not admissible" (p. 155), we should have acknowledged that he had rendered a great service to practical medicine and to afflicted humanity. We are, however, compelled to say, after carefully studying his book, that we can find no such indication fairly laid down in it. The muddle-headedness, and puzzle-headedness so characteristic of the whole volume, are fully as conspicuous in the essay of insanity as in the other parts of it; and, after turning the pages backwards and forwards half a score of times, we gave up the task in despair of finding what we so eagerly sought for. The following passages state clearly the mode in which Dr. Seymour administers the remedy; and one expression contained in the extract, as well as the title of the section formerly quoted, authorises us to conclude that it is in cases of *melancholia* that this mode of treatment is recommended; but we can find no clear definition of the forms or varieties in which it is especially applicable.

"The preparation which I have preferred, and, with two or three exceptions, I have always used, is the acetate of morphia; the mode of preparation the solution: forty drops of the solution which I have generally employed contain one grain of the alkaloid salt. It has generally been, in mild cases, my practice to begin by a quarter of a grain every night in solution; then, after a week, to increase this to half a grain: it has rarely in such cases been necessary to increase the dose beyond half a grain. In severe cases I begin with half a grain (twenty drops), and increase it speedily to a grain (forty drops); rarely, most rarely, beyond this dose. The medicine is given at bed-time, and only at bed-time, the period which is intended for sleep; but, it must be repeated, without the *intermission* of a single night, for several weeks in mild cases, for at the least three months in the most severe ones.—In some of these cases at first sleep is not produced; in very few *rest* is not produced. Slight nausea, and disturbance of the head, are felt the first few mornings, but in these cases almost always at first, and *always after* a short time, both sleep is procured and the waking hours are free from pain.

"The effect of the medicine is in precise analogy with what follows. Suppose a man toiling with professional anxieties, and with domestic cares, returns home, after a larger proportion than usual of the annoyances of his profession or calling, fatigued beyond his powers, wearied in mind. He returns to rest unhappy, discontented, inveighing against his lot, and what he considers to be his peculiar cares. He sleeps sound, and when about to rise in the morning, the sun streaming in at the windows, after a sound sleep, how does he look upon the evils of the preceding day? do they not lose a large portion of their affliction? does he not look in a totally different point of view at the very causes of distress which afflicted him the night before?

"And this is precisely what the effect of morphia, properly employed, effects in cases of melancholy mental derangement, but not once or twice, as would be the case in trifling distress. Hence it must be repeated regularly every night until the nervous system is soothed! Thus it requires weeks for the medicine to be repeated regularly, even without a single night's intermission, and the cure, as I shall show by examples, is the result." (pp. 159-61.)

"It is to be used in small quantities, regularly repeated, and never increased *beyond a certain point, whether taken for six weeks or six years!* I may say, with the greatest truth, I have never seen one single case where the treatment has succeeded, or where it has failed, that the smallest injury has been done, nor in a single case where the medicine has been continued beyond the time when health was re-established." (p. 163.)

Now, if Dr. Seymour could, indeed, "show us by examples," that "the cure" of melancholia generally is "the result" of such treatment, he should assuredly be our great Apollo in the treatment of insanity. He tells us, it is true, that during the last fifteen years, he has treated about ninety cases in this manner, and has thus cured by far the greater number.

"As fifteen years have elapsed......during which I have been anxiously watching the result of cases, especially of the recoveries, I can now with greater certainty point out the best mode of using this particular treatment. Upwards of seventy cases during that period of time have recovered; and I consider no case to be called a recovery unless two years at least of unabated health have elapsed since the treatment concluded. In nearly twenty cases the treatment has failed, or only given temporary relief." (p. 156.)

If there existed no source of fallacy in the grounds on which this statement is based, we should regard it as extremely important, notwithstanding our own experience and that of still higher practical authorities is far from being in accordance with it. But there is a most obvious source of fallacy in the premises, which completely invalidates the conclusions deduced from them,—at least in a large number of the cases: viz. *the very*

*long period during which the remedy was employed.* Every physician conversant with insanity knows that a considerable proportion of cases of melancholia, when observed in their earlier stages, recover within the period assigned by Dr. Seymour to the administration of his daily opiate viz. *months or years.* What right has Dr. Seymour to assume that a recovery that has ensued after months of his opiate treatment would not have ensued without it? Before we can be called on to admit this, we must not only have much more extensive statistics than he has given us of cases treated by opiates, but an equally large category of similar cases *treated otherwise, and with inferior results.* We are far from denying that the sedative treatment is beneficial in certain cases; and we admit that it was evidently so in some of the cases narrated in this book; but we feel it to be our duty strongly to caution our readers against being misled by Dr. Seymour's statements, as we are convinced that his treatment, if generally adopted, will not merely disappoint expectation, but be positively injurious. As an occasional remedy in the proper cases, and employed for a limited time, we regard it as valuable; but we believe the proportion of cases in which it is more valuable than other and commoner modes of treatment, is very small. It has, moreover, the great disadvantage attaching to all empirical and so-called specific remedies,—that, namely, of engendering and fostering a blind, uninquiring confidence, and sleepy satisfaction with our own proceedings, subversive of all rational treatment, and fatal to all improvement. We have ourselves, met with more than one lamentable example of this result, where patients had been pursuing this Seymourian treatment, not merely for months but years,—and not only in cases of melancholia, but in cases of mania and even of imbecility! Dr. Conolly, Dr. Hutcheson, and all the superintendents of large institutions whom we have consulted on this subject, object to the long-continued opiates recommended by Dr. Seymour; although all are in the constant habit of using them as occasional remedies, and with benefit in particular cases. The general feeling among the gentlemen of largest experience is, that the best sedatives and soporifics, speaking generally, are not to be found among drugs. The following are some of Dr. Conolly's views on sedatives:

"One of the best preparatives for good sleep," says Dr. Conolly, "is exercise in the open air; and an evening walk may act as a sedative. Rest at night is also more likely to be enjoyed by those who have not been irritated in the day, or before going to bed, or sent to bed cold or hungry, or thirsty and feverish. A kind manner, a little bread, sometimes a supper of bread and cheese and porter, or ale; or arrow-root with a little brandy; sometimes a glass of water, or a warm bath may be better than any sedative in the materia medica."*

"Any resident officer who frequents the wards late at night will learn that there are many of the patients to whom a cup of tea, or of broth, or of sago, or sometimes a glass of water, or sometimes a few kind words, act as an immediate sedative, and contribute also to the great end of producing satisfaction in the day, as well as solace in the night. I have repeatedly witnessed the good effects of such attentions, and have known violent and noisy patients tranquillized for the night by being allowed water to wash their hands and face, and water to drink, and by having the bed made up afresh, and a cool dress to put on, and a cheerful good-night wished to them by kind-hearted attendants. To withhold all this, as if it were so much weak indulgence of the capricious, is to treat the insane as criminals, not as patients, and cannot be too strongly reprobated."†

* The Construction and Government of Lunatic Asylums, p. 99. † Ibid. p. 103.

We believe that Dr. Seymour's observations on "the effect of travelling, or what is called variety," in melancholia are correct.

"Thus it is, and thus it has been forced on my mind, in a great number of examples, both of men and women, that change of place and variety is eminently injurious as a means of cure, until the melancholy hallucinations are completely at an end." (p. 179.)

Under the head of employment, Dr. Seymour tells us that "the fact of being able and willing to work, is a *prima facie* evidence that their mental disease is curable." (p. 181.) This idea we suppose is derived from his experience as a commissioner of lunacy; but we believe that at the time he held that office opportunities of employment were rarely afforded. He also speaks of "compulsory employment;" but who ever recommended it?

We entirely demur to the recommendation (p. 184) of applying leeches to the os uteri in the case of unmarried women. We believe that leeches applied to the sacrum or pubes, in suppressio mensium, and in hysterical mania, are often as efficacious as when applied to the os uteri; and in every respect less objectionable. These unfortunate women, the hysterical especially, are submitted to many needless and cruel measures, and in Parisian practice, to many villanous methods of treatment, of which the effects are always lamentable, and, we believe, not seldom fatal.

Dr. Seymour makes some just and important observations on the relative value of seclusion in one's own house, in private houses, and in asylums. We are, however, astounded at the following statement: "I must, however, in common justice, premise that during my period of duty as a commissioner of lunacy, I never witnessed one case of *cruelty*. The greatest care was taken, the most assiduous, nay, the most invidious (to innocent people) inquiries were made." We think it shows how little a commissioner may know of what passes in the sphere of his duties. It is discredited by the records of numerous inquests, and by the state in which, even now, patients are brought to public asylums from private asylums. We are disposed to agree with Dr. Seymour in demurring to the truth or justice of the following propositions:

"1st, then, it has become a system, partly from tradition, partly from usage, no physician taking it up, that no one can recover except when removed from his family.

"2d. That it is not only desirable, but necessary, that the patient should not see his friends, except on visits very few and far between, during his confinement.

"3d. An extreme disinclination to let the patient have a trial; that is, to be removed home." (p. 218.)

In his examination of the first of these objections, he says:

"Something is to be attributed to the indisposition to undertake a difficult and unmanageable case by men actively engaged in the profession. They answer, 'really this is a case I am not accustomed to; you must send to one of those gentlemen who make it their exclusive study.' Now, this is precisely what I think to be erroneous: it is because they make it their exclusive study, that I think they are less good judges than they otherwise would be of the *cure* of such cases." (pp. 218-19.)

It is surely a strange doctrine this, that persons should be worse judges of any matter in proportion to their experience, observation, and reflection! What will Dr. Conolly, Dr. Sutherland, or Sir Alexander Morison say to this doctrine? On the same principle of *lucus a non lucendo,* we do not doubt that Dr. Seymour considers himself much better qualified to give an accurate diagnosis in diseases of the chest, and consequently to treat them more scientifically, than Sir James Clark, Dr. Latham, Dr. Williams, or Dr. Walshe.

IV. The last essay in the volume, that on SCIATICA, extends only to thirty-six pages, and is by much the best of the four. Although it contains nothing that will be new to any practical physician of experience, we think the young practitioner may read it with advantage. It has qualities which the reader of the other essays would hardly expect to find in it. Though abounding in errors of style, it is written clearly and intelligibly enough ; the thoughts even run in natural sequence ; and, what is still more remarkable, the disease and its treatment are studied and expounded in a rational pathological manner, and not merely empirically. With some of the pathology we cannot agree ; but we are disposed to regard the practice recommended, as that which most men of experience will be disposed to sanction.

The all-important point in setting about the treatment of a case of sciatica, is to know what the disease is we are called on to treat, as other diseases of an entirely different kind are sometimes confounded with it, while sciatica, even that properly so called, varies greatly in its nature in different cases. Dr. Seymour notices the distinctive symptoms between sciatica and the two affections with which it is most apt to be confounded, viz., diseases of the hip-joint and disease of the kidney.

"Having thus ascertained that pain in the course of the sciatic nerve is neither connected with incipient disease of the hip-joint, nor dependent on irritating secretion in the urine, the physician must proceed to inquire whence it proceeds, and whether it is idiopathic, that is, depending on the state of the nerve itself, or symptomatic of disorder of the stomach, or of syphilitic taint, or, finally, whether it is the first indication of disease in the brain itself. The first attention should be paid to the general health of the patient. Is he robust, living well, taking much food and little exercise, having enjoyed good health for many years ? If this be the case, it is more than probable that a brisk succession of purgatives will cure the disease; such pain, if on the right side, occasionally depending on distension and obstruction in the caput coli ; or, if it has succeeded to cold blasts, upon the body being in the state mentioned, then cupping, followed by blisters, will be useful to relieve the pain ; or the vapour-bath, or diaphoretic remedies carefully continued, will cure the case." (pp. 229-30.)

When the disease occurs from over-exertion in walking, in a person unaccustomed to exercise, and of a weak disordered habit, "the painful affection of the nerve is to be soothed by sedatives, while the muscular force of the body is upheld by nourishment and strengthening medicines." (p. 230.)

"Is the patient a gouty man ? Has he suffered from gout ? If he has, and this painful affection occurs in an interval of the disease, it may fairly be classed with other cases of erratic gout, and treated accordingly. The various preparations of colchicum will be of great importance ; but in the cases which I have witnessed (and they were very severe), quinæ sulphas in large doses given in the day time, with thirty drops of the wine of colchicum in a draught at night, were most effectual ; but neither separately produced permanent relief." (pp. 231-2.)

Sciatica, like other forms of neuralgia, may arise from many other disorders of the system, and must be treated accordingly. But it exists also in an idiopathic form, "without any discoverable cause at a distance."

Dr. Seymour is "inclined to be of the opinion" of Cotunnius—of whom he says in his own droll manner, "He lived to a great age—*hence* his work may be looked upon as an *ancient* classic,"—that the disease in certain cases "arises principally from effusion of fluid into the sheath of the nerves;" and he thinks that acupuncture, which he strongly recommends, may act by "setting free the fluid." Dr. Seymour passes in review all the remedies, in ordinary use for tic douloureux, but tells us nothing new; and tells us much less and less satisfactorily than we are told by Dr. Hunt and others who have written *ex professo* on the disease. But the most important part of Dr. Seymour's observations is that which refers to Sciatica, "as symptomatic of disease of the brain." He says he has seen five cases of this sort, in some of which, however, the pain was seated in the brachial nerves; and he has given notes of two. In both of these, vomiting supervened, and is considered by Dr. Seymour as indicative of the cerebral cause. As, however, Dr. Seymour had, *more suo,* prescribed sedatives in these cases, it was doubtful whether the vomiting might not be attributed to them. In common with other physicians, we have ourselves met with similar cases, and have been more than once not merely puzzled but at first mistaken, as to the true character of the disease. We are sorry to find that Dr. Seymour gives us no help towards the more accurate discrimination of such cases in future.

Although Dr. Seymour may not be inclined to follow any advice we may give, we shall, nevertheless, advise him to be content with what he has already done, and eschew printing for the future. He may rest assured that such is the most friendly advice that could be given to him. But if, as coming from us, he should (wrongly) imagine it not to be friendly, we would remind him that, like a nauseous medicine, it might yet be beneficial—*fas est ab hoste doceri.* If, however, he is resolved to carry out the plan he has announced, giving us, according to the positive declaration of the title-page, one more volume, or, according to the conditional threat in the preface, two more volumes, we trust that he will, at least, take means to convey to us his future Thoughts in a form less offensive to good taste and less obnoxious to criticism, than the form in which they reach us in the present. From some passages in the volume before us—and we have extracted the best of these—we have a suspicion that Dr. Seymour could write better if he chose; but whether he can not, or can and will not, we do hope that, for the sake of his order and the character of the medical literature of the country, as well as out of regard to the interests of those he desires to instruct, if not for his own reputation, he will not permit the succeeding volume or volumes to be like the present. If not aware of the fact, we take the liberty of informing him, and on the unimpeachable authority of the 'Times' advertising columns, that there are hundreds of "men of letters" in this metropolis, who will be delighted to see his or any other person's MSS. safely through the press, in creditable and workman-like manner. Or should he prefer a less formal and more economical proceeding, we doubt not that he will find among the present pupils of his old hospital, more than one who have not yet forgot their grammars, and who will be proud to be allowed to translate his galimatia into the vernacular tongue.

